# Intelligent delignification: leveraging explainable AI for ozone transport modeling and optimization

**DOI:** 10.1038/s41598-025-28638-7

**Published:** 2025-12-29

**Authors:** Muhammad Rizwan, Muhammad Ahmad Khan, Sharifullah Khan, Khurram Shahzad Baig, Aitizaz Ali, Mohamed Shabbir Abdulnabi, Maqbool Khan

**Affiliations:** 1 School of Computing Sciences, Pak-Austria Fachhochschule: Institute of Applied Sciences and Technology, Haripur, 22650 Pakistan; 2 Department of Chemical and Energy Engineering, Pak-Austria Fachhochschule: Institute of Applied Sciences and Technology, Haripur, 22650 Pakistan; 3 Software Competence Center Hagenberg, Softwarepark 32a, Hagenberg, 4232 Austria; 4 American University of Bahrain, Riffa, 0942 Bahrain; 5 School of Technology, Asia Pacific University of Technology & Innovation (APU), 57000 Kuala Lumpur, Malaysia

**Keywords:** Delignification, Lignin, Machine Learning, XAI, Modelling, SHAP, Energy science and technology, Engineering, Environmental sciences, Materials science, Mathematics and computing

## Abstract

Biomass is mainly composed of cellulose, hemicellulose, and lignin, where lignin is almost one-third of the amount of biomass. Lignin is removed from the biomass matrix because its complex, recalcitrant structure acts as a physical and chemical barrier, impeding the accessibility of reagents and enzymes to cellulose and hemicellulose. Lignin sterically hinders hydrolysis and can also non-productively bind enzymes, thereby reducing overall efficiency in biofuel production. Several techniques are used to remove lignin from the biomass matrix, where ozonation is the most novel, emerging, and ecofriendly technique. This study investigates the application of machine learning (ML) techniques to predict lignin removal efficiency during ozonation pretreatment an emerging and eco-friendly delignification method. Experimental data from ozonation-based lignin removal were used to train and evaluate 19 regression models using the PyCaret framework. Among these, the Extra Trees Regressor demonstrated the highest predictive accuracy for delignification outcomes. Feature importance was further interpreted using SHapley Additive exPlanations (SHAP) to quantify the contribution of each process variable. The results reveal that ML models can effectively capture the complex relationships governing ozonation-based delignification, offering valuable insights into optimizing operational parameters. This work highlights the potential of ML as a predictive and interpretative tool in chemical engineering, paving the way for more efficient, data-driven approaches to biomass valorization and sustainable biofuel.

## Introduction

The usage of liquid fuel, including both fossil fuels and renewable biofuels, has increased significantly during the last 20 years. To provide energy, lignocellulosic biomass wastes are usually converted into liquid fuels^1,2^. Animals and farms are the original creators of these waste materials. A wide variety of materials can be used as biomass waste, including wood or forest residues, food processing and livestock agricultural byproducts, and water treatment plant effluent^3^. Cellulose, hemicellulose, and lignin are the main components of agricultural (biomass) waste. In terms of structure, lignin acts as a mediator between cellulose and hemicellulose by filling the gaps in the cell wall. Lignin is chemically cross-linked with cellulose and bonded covalently to hemicellulose^4,5^. While cellulose and hemicellulose are the primary substrates for conventional biofuel production, lignin is also a valuable aromatic polymer with significant potential for advanced biofuels and value-added chemicals. In current industrial practice, lignin is often removed during pretreatment to enhance saccharification efficiency; however, its utilization is an emerging focus in sustainable biorefinery research^6^.

Physical, physiochemical, chemical, and biological techniques are the four main pretreatment types^7^. Reducing particle size is the main goal of these pretreatments because it increases the surface area of the substrate and degrades crystallinity and polymer chains^8,9^. A large amount of energy is consumed for physical preparation, and a large investment in the required mechanical equipment is a major downside^10^. Another option is to use chemical pretreatment, which might include acids, alkalis, ionic liquids, or deep eutectic solvents^11^. Chemical pretreatment is successful, but it is limited to the use of solvents that are expensive, harmful to the environment, and potentially very combustible^12^. On the other hand, cellular and enzymatic biological pretreatment is an attractive environmentally friendly alternative that uses less energy and typically requires fewer harsh chemical additives compared to conventional pretreatment methods, although the formation of inhibitory compounds can still occur^13^. Physiochemical pretreatments come with their own set of pros and cons. Some examples are ozonation, wet oxidation by hydrogen peroxide, carbon dioxide (CO_2_) or steam explosion, liquid hot water, and ammonia fiber explosion (AFEX). Due to its lower production of hazardous waste compared to traditional procedures, ozonation pretreatment has attracted great study attention^14,15^.

Due to the slow response rate with dry wheat straw, which might lead to problems with ozone decomposition, previous research has shown that it is beneficial to wet the straw before ozonation. Theoretically, the increase in the accessibility of ozone to the interior cells is due to the swelling of the wheat straw from absorbed water. The intricacy of the problem suggests that a technique would be useful that can both forecast the benefit of lignin removal and shed light on the relative significance of the components involved in this process. Machine learning (ML) techniques, which are data-driven, work well for this type of predictive modeling^16^. Data collected from experiments was used to train the models where statistical models and computing methodology was used for the analysis and interpretation of complex datasets of the underlying chemical processes and ML in the context of chemical engineering^17–19^. Experts in this area can improve their working processes, get insights, and make data-driven decisions by employing sophisticated mathematical methodologies^20^. Predictive models may be more easily developed with the help of ML when used in chemical engineering. This leads to a greater efficiency and a better knowledge of the complex interactions inside chemical processing systems^21–23^. This modeling effort considers the correlations between structure and properties employed in this study. To bridge the gap in delignification experimental data and result prediction using an easy-to-use model for lignocellulosic matrix samples. Using lignocellulosic matrix conditions in delignification process.

This work aims to develop ML models that can forecast the amount of lignin removed. The urgent demand for efficient and precise methods for predicting biomass processing is being met by this attempt, which constitutes a new addition to chemical engineering. An innovative aspect of this work is the incorporation of explainability into ML models that are designed to investigate the process of lignin extraction from lignocellulosic materials. This integration uses the Explainable Artificial Intelligence (XAI) approach through SHapley Additive exPlanation (SHAP) techniques to make these ML models more trustworthy and useful^24,25^. Therefore, the study goes beyond providing accurate prediction models. It also gives practical insights that may be used to further lignin removal research in both academia and industry.

## Results and discussion

### Data analysis

Among data scientists and ML experts, Python stands head and shoulders above the others. TensorFlow, Scikit Learn, Matplotlib, and Pandas were utilized^26^. The data frame was created, and the data was loaded using Pandas. The data visualization tool Matplotlib is utilized. Different regression algorithms may be implemented with PyCaret, a traditional ML package.The data frame presents the information obtained from the 51 samples used in the laboratory studies.

**Fig. 1:**
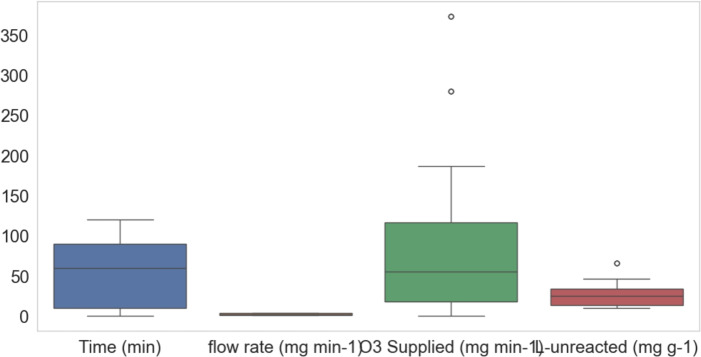
Boxplot of features. The diamonds represent outlier data points that fall outside the interquartile range (IQR) for each feature, indicating values that are significantly higher or lower than the majority of the data.

**Fig. 2:**
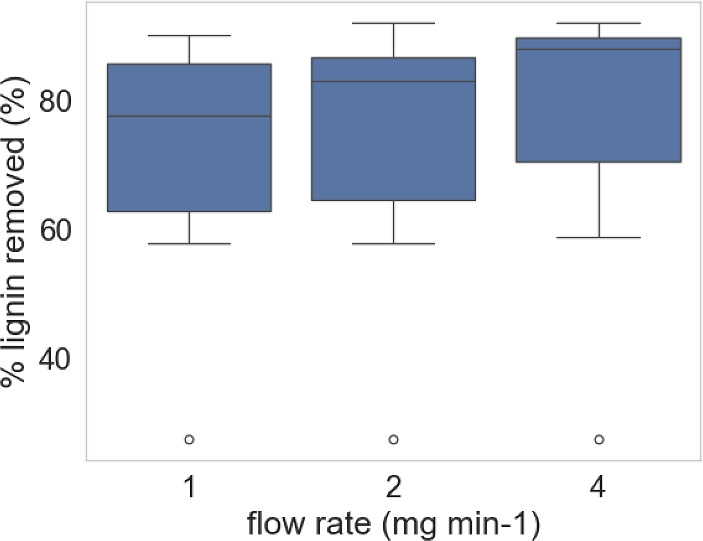
Graphical representation of lignin removal percentages obtained from experimental data. The diamonds represent individual experimental data points that lie outside the typical range of values (outliers) for each flow rate condition.

Figure 1 illustrates the boxplot of the experimental features. The diamonds represent outlier data points that fall outside the interquartile range (IQR) for each feature, indicating values significantly higher or lower than the majority of the data. As shown, features with larger numeric ranges naturally exert a greater influence on the model; therefore, normalization was applied to standardize the data. The characteristic to be normalized is represented by $\sigma$ (standard deviation), while the average is denoted by $\mu$ (mean). The dataset was then divided into training (80%) and testing (20%) subsets to ensure proper model evaluation. Figure 2 presents the graphical representation of lignin removal percentages obtained from experimental data, where the diamonds represent individual experimental outliers for each flow rate condition.

**Fig. 3:**
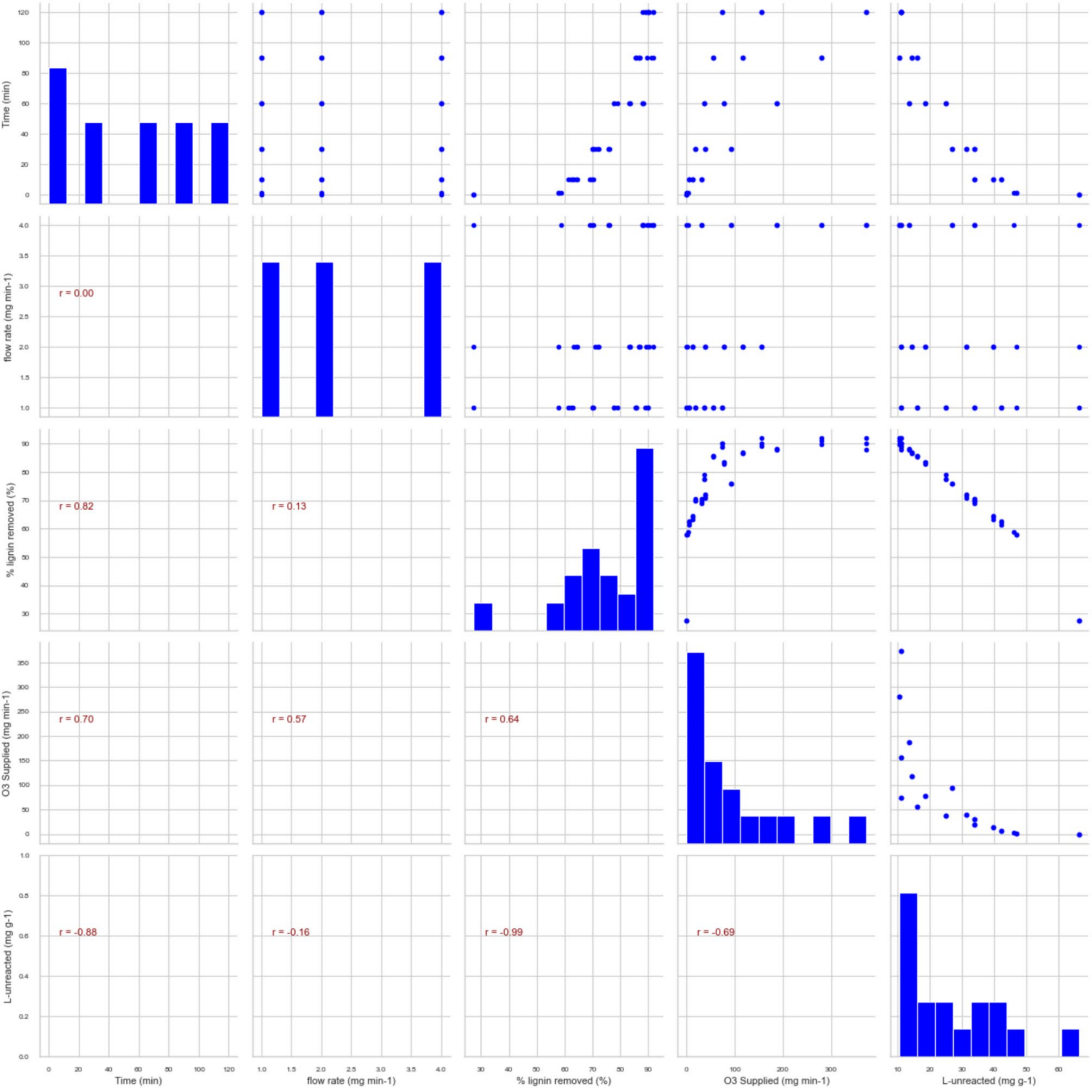
Graphical co-relationship of data generated from the laboratory experiments.

A pair plot or correlation matrix is a powerful visualization tool used to explore the relationships between multiple variables in a dataset. The plot displays five key variables: time (min), flow rate ($\hbox {mg min}^{-1}$), % of lignin removed, O_3_ ($\hbox {mg min}^{-1}$) supplied, and L-unreacted ($\hbox {mg g}^{-1}$) (likely referring to unreacted lignin) as shown in Fig. 3. Each variable is plotted against every other variable, allowing for a comprehensive examination of the interactions within the dataset.

The diagonal of the plot shows histograms for each variable, illustrating their distribution. These histograms help us understand the spread and central tendency of the data for each, displayed in some scatter plots. The value of r ranges from −1 (indicating a perfect negative correlation) to +1 (indicating a perfect positive correlation), with 0 signifying no linear correlation. In the upper triangle of the matrix, the scatter plots show how each variable relates to the others, with the corresponding correlation coefficients included in some cells. For example, a strong positive correlation ($r = 0.82$) is observed between the time and % of lignin removed, suggesting that longer reaction times lead to higher lignin removal efficiency. Similarly, a moderate positive correlation ($r = 0.70$) between Time and O_3_ supplied indicates that more ozone is supplied as the reaction time increases. However, the scatter plots also reveal weaker correlations, such as between flow rate and other variables, where the correlations are low ($r = 0.00$, $r = 0.13$, etc.), suggesting that flow rate changes may not significantly impact the other variables in this dataset.

The lower triangle also contains scatter plots, which mirror those in the upper triangle but with different orientations. The strong negative correlation ($r = -0.99$) between % of lignin removed and L-unreacted is particularly noteworthy. This near-perfect negative correlation is expected because as more lignin is removed, less unreacted lignin remains. Similarly, the scatter plot between O_3_ supplied (expressed as mg O_3_ $\hbox {min}^{-1}$) and L-unreacted shows a moderate negative correlation ($r = -0.69$), implying that increasing the amount of ozone supplied generally resulted in less unreacted lignin.

**Fig. 4:**
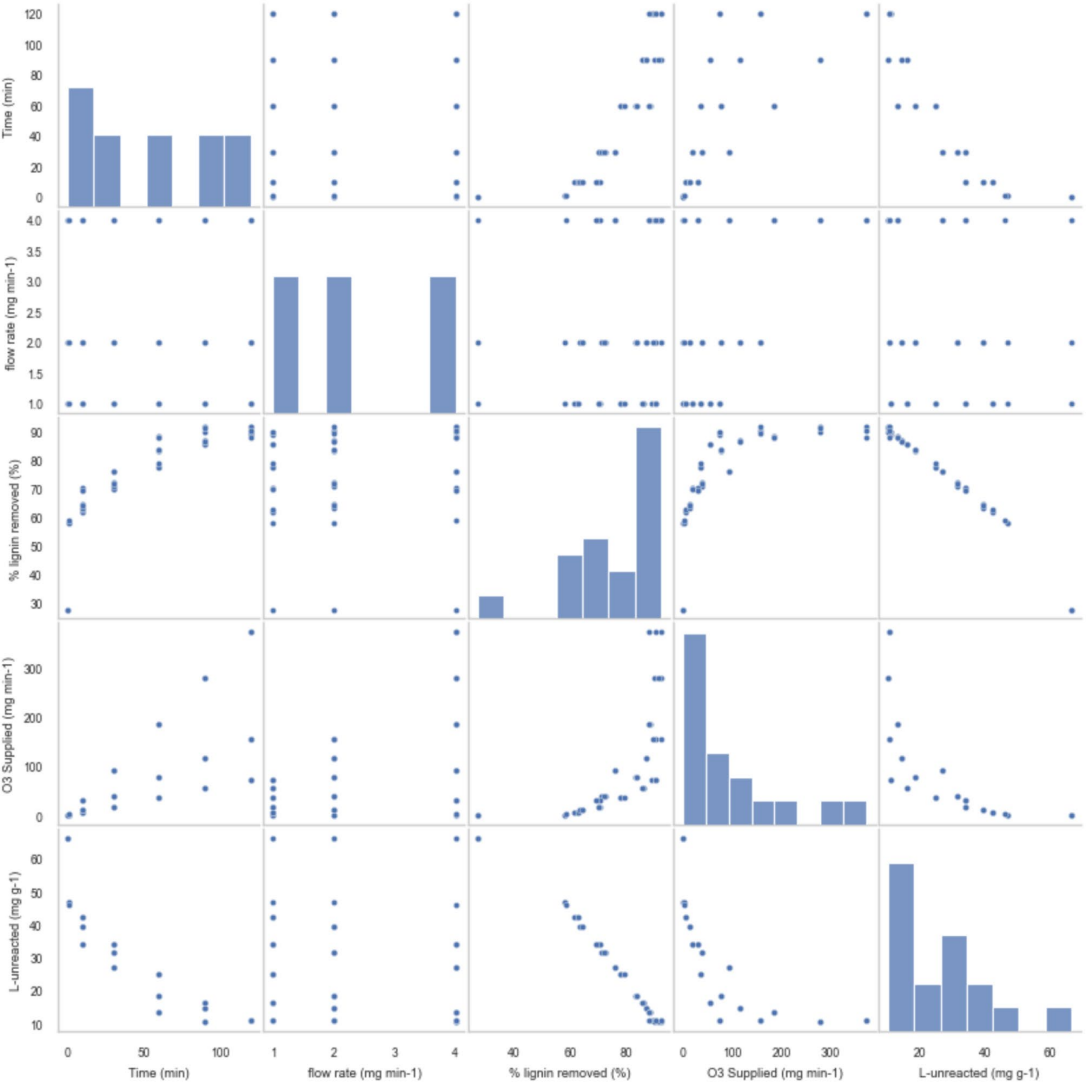
Pair plot comparing the relationship among all the variables.

This pair plot offers valuable insights into the relationships between the key variables involved in the ozonation process. It highlights the importance of time and ozone supply in enhancing lignin removal efficiency while indicating that flow rate may have a limited impact. The strong correlations provided in this plot Fig. 3 provide a foundation for further optimization efforts, guiding the focus towards variables that significantly influence the outcome of the process.

To visualize the relationships between several variables in a dataset, specifically time, flow rate, % of lignin removed, O_3_ supplied and L-unreacted ozone. Each variable is plotted against every other variable to assess potential correlations and patterns as shown in Fig. 4. The diagonal of the plot features histograms for each variable, showing the distribution of values. For instance, the histogram for time illustrates the spread across various time intervals, while the % lignin removed histogram highlights a more varied distribution, with a notable concentration around higher removal percentages.

The off-diagonal plots are scatter plots that demonstrate how pairs of variables relate to each other, offering a glimpse into potential linear or non-linear relationships. For example, the scatter plot between time and % of lignin removed suggests a positive relationship, indicating that longer reaction times are associated with higher lignin removal. The flow rate variable shows a limited spread across its scatter plots, implying that it may not vary significantly across the dataset or strongly influence other variables. Meanwhile, the relationship between the % of lignin removed and O_3_ supplied appears curved, suggesting an optimal ozone supply level beyond which further increases may not significantly improve lignin removal. Additionally, a strong inverse relationship is observed between the % of lignin removed and L-unreacted, which is expected since higher lignin removal naturally corresponds to lower amounts of unreacted lignin.

The pair plot is symmetric around the diagonal, meaning that the relationships depicted in the upper triangle mirror those in the lower triangle. For example, the relationship between % of lignin removed and L-unreacted ozone appears in both the upper and lower triangles. This symmetry highlights the consistent relationships between variables regardless of the order in which they are plotted.

The pair plot provides a comprehensive visual summary of the interactions between these variables in the context of the ozonation process. It helps identify key relationships, such as the strong positive correlation between time and % of lignin removed and the strong negative correlation between % of lignin removed and L-unreacted ozone. The plot also indicates that variables like flow rate may not significantly impact the outcomes, as evidenced by the relatively flat or sparse scatter plots involving flow rate. Overall, this visualization offers valuable insights into which factors most significantly influence lignin removal efficiency in the dataset.

**Fig. 5:**
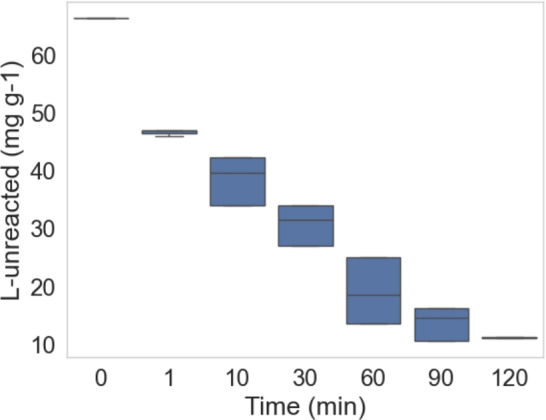
Box plot showing lignin removal efficiency increases with longer reaction time.

A box plot that illustrates the relationship between reaction time and the amount of unreacted lignin during a set of experiments. The x-axis represents the reaction time, ranging from 0 to 120 minutes, while the y-axis displays the quantity of unreacted lignin as shown in Fig. 5. The different box plots correspond to various time intervals, providing insight into how the amount of unreacted lignin changes as the reaction progresses.

At the initial time point (0 minutes), the box plot appears as a flat line at a higher value, indicating that no lignin has reacted. As time increases, there is a notable decrease in the amount of unreacted lignin. The downward shift in the box plots over time reflects this trend. Each box represents the interquartile range (IQR), which shows the middle 50% of the data, and the horizontal line within each box indicates the median value of unreacted lignin at that specific time. The whiskers extending from the top and bottom of each box illustrate the spread of the data, with any points outside this range considered outliers.

The overall trend depicted by the box plots shows a consistent decrease in the amount of unreacted lignin as reaction time increases. This aligns with the expectation that longer reaction times would lead to more complete lignin removal. By the 60-minute and 90-minute marks, the amount of unreacted lignin is significantly lower, although some variation still exists. However, there are no visible outliers in this plot, suggesting that the data remains relatively consistent across different time intervals.

This box plot effectively demonstrates how reaction time influences the amount of unreacted lignin. The general downward trend in median lignin values, as time progresses, indicates a more efficient lignin removal process over a longer reaction period as in Fig. 5.

**Fig. 6:**
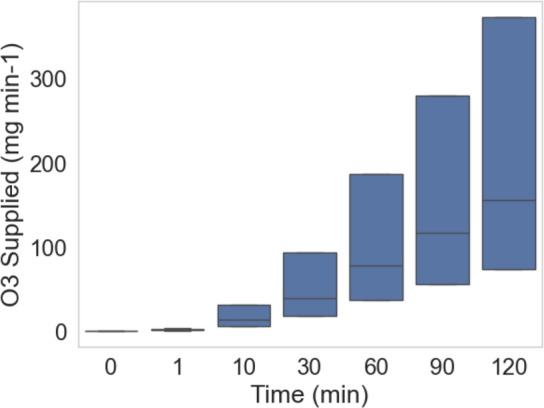
The median values of O_3_ supplied over time.

The relationship between time and the amount of O_3_ supplied is shown in Fig. 6. The x-axis represents time, with increments ranging from 0 to 120, likely in units of minutes. The y-axis represents the quantity of O_3_ supplied. The data points are visualized using box plots, where the box represents the IQR, encompassing 50% of the data, with the median value marked within the box. The whiskers extend to capture most of the data, excluding outliers represented by individual points.

**Fig. 7:**
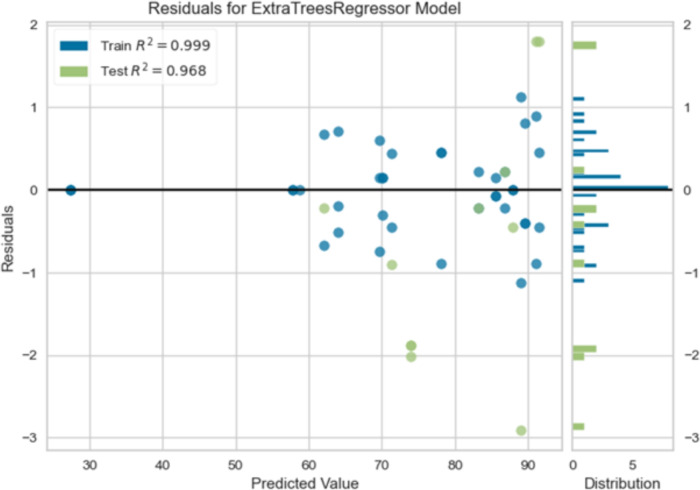
The median values of O_3_ supplied over time.

Overall, Fig. 6 shows a clear increasing trend in O_3_ supplied over time. The median values steadily rise with each time point, indicating a consistent upward trajectory. The variation in O_3_ supply, as Overall, Fig. 6 shows a clear increasing trend in O_3_ supplied over time. The median values steadily rise with each time point, indicating a consistent upward trajectory. The variation in O_3_ supply, as represented by the box lengths, also appears to increase over time, suggesting potentially greater variability in the later stages.

The diagram presents the residuals for an extra trees regressor model, visualizing the difference between the predicted and actual values. In Fig. 7 the x-axis represents the predicted values, ranging from approximately 30 to 90, while the y-axis represents the residuals. The plot includes two types of data points: train residuals (blue) and test residuals (green). A horizontal black line is drawn at the zero residual value as a reference point.

The distribution of residuals is displayed in the right panel, showing the frequency of residuals within specific ranges. The blue bars represent the train residuals, while the green bars represent the test residuals. This visualization helps to understand the spread and distribution of errors. The R-squared values for the train and test sets are provided in the legend: 0.999 for the train and 0.968 for the test.

**Fig. 8:**
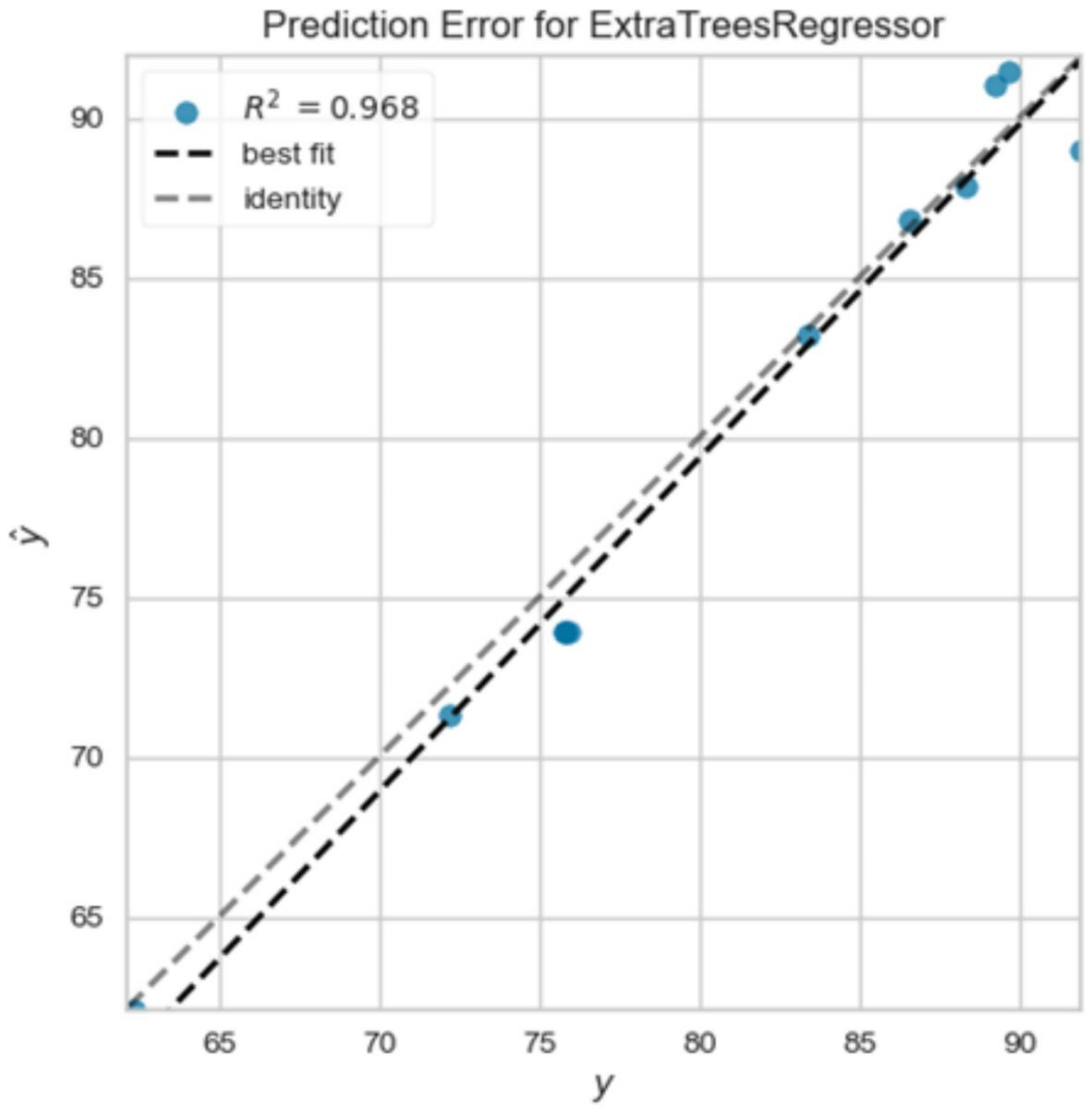
True values with respect to predicted values.

In Fig. 8 presents the prediction error for an extra trees regressor model, visualized through a scatter plot. The x-axis represents the true values (y), while the y-axis represents the predicted values (ŷ). Each blue dot signifies a data point, showcasing the relationship between the actual and predicted values. The dashed black line, labeled “best-fit”, represents the ideal scenario where the predicted values perfectly align with the true values. The gray dashed line, labeled “identity”, represents a perfect prediction, where the predicted values are identical to the true values.

Figure 8 shows that the R-squared value of 0.968 indicates the model’s performance. A higher R-squared value suggests a better fit, implying that the model can explain a significant portion of the variance in the data. In this case, the R-squared value of 0.968 suggests that the model explains 96.8% of the variability in the data.

The distribution of the blue dots around the “best-fit” line reveals the model’s accuracy. Points closer to the line indicate better predictions, while points farther away represent larger errors. The overall pattern suggests that the extra trees regressor model generally performs well, with most predictions falling close to the ideal line.

**Fig. 9:**
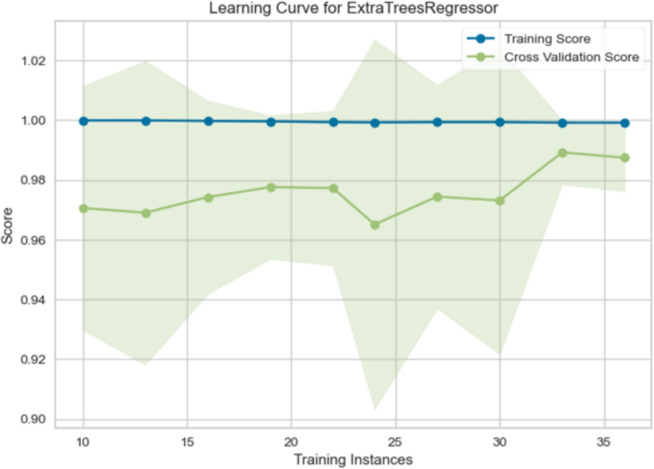
The model’s accuracy.

The learning curve for an extra-trees regressor model, visualizing the model’s performance as the training set size increases, is shown in Fig. 9. The x-axis represents the number of training instances, ranging from approximately 10 to 35. The y-axis represents the score, which likely measures the model’s accuracy or performance metric. Two lines are plotted: the training score (blue) and the cross-validation score (green). The shaded areas around the lines represent the standard deviation of the scores. The training score remains consistently high and relatively flat throughout the range of training instances. This indicates that the model learns the training data very well and quickly reaches its maximum performance in the training set. The cross-validation score shows an initial increase as the number of training instances increases, indicating that the model’s generalization performance improves with more data. However, after a certain point, the cross-validation score plateaus, suggesting that adding more training data does not significantly enhance the model’s ability to generalize to unseen data.

### Machine learning models results

The following evaluation matrices were used to assess the performance of 19 classic ML models: mean absolute error (MAE), mean squared error (MSE), root mean squared error (RMSE), standardized root mean squared ($R^2$), root mean squared logarithm error (RMSLE), and mean absolute percentage error (MAPE). The results can be seen in Table 1. Extra trees regressor outperforms all other traditional models with the best $R^2$ (0.9874), MAPE (0.01), and RMSE (0.907). Extreme gradient boosting, least angle regression, and decision tree regressor are the other three best models. Extreme gradient boosting has an RMSE of 1.0591, MAPE of 0.0125, and $R^2$ of 0.9763. Least angle regression has an RMSE of 1.5796, MAPE of 0.0233, and $R^2$ of 0.9758.

**Table 1:** Performance Metrics of Various Regression Models.

Sr. No	Model	MAE	MSE	RMSE	R^2^	RMSLE	MAPE
1	Extra Trees Regressor	0.7542	0.9400	0.9070	0.9874	0.0117	0.0100
2	Extreme Gradient Boosting	0.9270	1.5293	1.0591	0.9763	0.0141	0.0125
3	Least Angle Regression	1.3700	3.1228	1.5796	0.9758	0.0282	0.0233
4	Decision Tree Regressor	0.8910	1.5474	1.0423	0.9754	0.0131	0.0114
5	Random Forest Regressor	1.4340	4.5626	1.7716	0.9744	0.0339	0.0266
6	Bayesian Ridge	1.4260	3.6382	1.7192	0.9727	0.0316	0.0248
7	AdaBoost Regressor	0.9570	1.9891	1.1505	0.9727	0.0147	0.0124
8	Linear Regression	1.4225	3.6164	1.7131	0.9726	0.0310	0.0245
9	Huber Regressor	1.4805	4.3982	1.8840	0.9689	0.0395	0.0288
10	Ridge Regression	1.6558	6.1640	2.1663	0.9685	0.0493	0.0356
11	Gradient Boosting Regressor	1.2678	7.6433	1.7647	0.9650	0.0279	0.0180
12	Lasso Regression	1.8392	9.7410	2.6172	0.9595	0.0612	0.0430
13	Lasso Least Angle Regression	1.8392	9.7410	2.6172	0.9595	0.0612	0.0430
14	Passive Aggressive Regressor	1.8735	5.9569	2.3074	0.9537	0.0442	0.0332
15	Orthogonal Matching Pursuit	2.1815	8.1879	2.6787	0.9466	0.0566	0.0432
16	CatBoost Regressor	2.3628	33.8296	3.5810	0.9242	0.0807	0.0631
17	Elastic Net	4.0149	47.4800	5.4133	0.8282	0.1131	0.0921
18	K Neighbors Regressor	4.0730	56.7437	5.7279	0.8043	0.1212	0.0992
19	Light Gradient Boosting Machine	13.6749	303.9522	15.936	−0.5799	0.2572	0.2598

### Interpretable models and interpretation of features importance

**Fig. 10:**
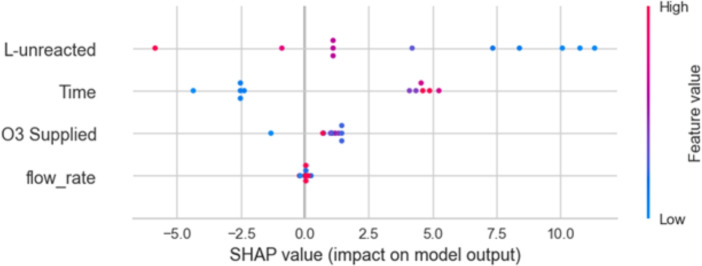
Interpretation of features importance.

SHAP values are used for various features in a model, likely predicting a specific outcome related to chemical or biological processes. SHAP values quantify the impact of each feature in this model’s output, including L-unreacted, Time, O_3_ Supplied, and flow rate. The x-axis represents the SHAP value, with negative values indicating a decreasing impact on the output, and positive values indicating an increasing impact as shown in Fig. 10.

The color intensity of the dots represents the magnitude of the feature value, with darker colors indicating higher values. Each horizontal row represents a different feature. For example, the row for L-unreacted shows that higher values of L-unreacted generally have a negative impact on the model output, as indicated by the predominantly red dots. O_3_ Supplied and L-unreacted are the most influential features in the model, as their dots are more spread out and cover a wider range of SHAP values. Time and flow rate seem to have less impact on the model’s output, with their dots clustered closer to the center.

### Discussion

The results demonstrate that the Extra Trees Regressor achieved the highest predictive accuracy among the 19 evaluated ML models, as indicated by its superior $R^2$ (0.9874), lowest RMSE (0.907), and minimal MAPE (0.01). These findings suggest that ensemble-based models can effectively capture the complex nonlinear interactions between process variables involved in lignin degradation. This aligns with previous studies that reported strong performance of ensemble methods, such as Random Forest and Gradient Boosting, in modeling chemical and environmental systems due to their robustness and ability to handle multicollinearity.

The SHAP analysis further revealed that $\hbox {O}_3$ supplied and L-unreacted were the most influential features affecting lignin removal efficiency. This indicates that ozone dosage directly determines the oxidative breakdown of lignin molecules, while unreacted lignin serves as a reliable indicator of reaction completeness. The relatively lower impact of flow rate and reaction time suggests that these parameters may have already reached an optimal operational range in the experimental design.

From an application perspective, these results highlight the potential of ML-assisted optimization in lignocellulosic biomass pretreatment. By accurately predicting lignin removal efficiency, such models can help minimize chemical usage and reaction time, leading to more sustainable and cost-effective biorefinery operations. This supports the broader goal of integrating AI and process analytics into biomass conversion technologies to enhance environmental performance.

However, the study has certain limitations. The dataset size (51 samples) limits the statistical power and generalizability of the model. Furthermore, the experiments were conducted under controlled laboratory conditions, which may differ from industrial-scale variability. Future work should focus on expanding the dataset, incorporating additional reaction variables (e.g., pH, temperature), and validating model performance on pilot-scale systems.

Overall, the combination of experimental and ML analyses demonstrates a powerful framework for understanding and optimizing lignin removal processes. The results contribute valuable insights into the interactions between time, ozone dosage, and lignin reactivity, providing a foundation for developing intelligent, data-driven process control strategies in biorefinery applications.

## Methods

To predict lignin removal from lignocellulosic matrices, an Artificial Intelligence (AI) approach is used that incorporates explainability into ML models. At the first stage, a set of ML models was built and used to forecast the removal of lignin under different experimental settings. Next, the results of the model’s prediction and related attributes to improvement in explainability were looked. To provide a comprehensive analysis of the factors influencing lignin removal efficiency, including flow rates($\hbox {mg min}^{-1}$), ozone (O_3_) ($\hbox {mg min}^{-1}$) concentration, reaction time (min), and L-unreacted ozone ($\hbox {mg g}^{-1}$), so that future studies can make the most efficient use of available resources. This is accomplished by utilizing popular explainability approaches, such as SHAP^27,28^.

### Materials

A farmhouse in Toronto provided two bundles of wheat straw (Tritium sativum). The wheat straw was washed with deionized water. A soaking solution containing 1% sodium hydroxide (Sigma Aldrich, St. Louis, MO, USA) and a hydrolysis solution containing 72% sulfuric acid (H2SO4) were also utilized. The straws were ground using a Comfort machine, specifically a model SM100 from Retsch Inc. in Haan, Germany. To dry the wheat straw residues, Labline Inc. supplied an oven of type No. 3605 with 1200 watts. The process of creating ozone from oxygen was carried out using a GL-1 generator (PC1-WEDCO). A spectrophotometer model S500 from Biochrom Libra that has an ultraviolet (UV) light detector was used to measure the lignin content^29^.

### Experimental setup

The ozonation reaction was tested with three different reactor configurations. Each reactor was linked to an ozone generator that could produce pure oxygen ozone concentrations ranging from 6.5 to 65.5 mg/L. A column reactor using a fluidized-bed technology with dimensions of 3.5 $\times$ 25 cm was the simplest design. Both the two-phase solid-gas reactor and the three-phase solid-water-gas reactor relied on fluidization concepts. To monitor the input, the stainless steel two-phase solid-gas reactor had control valves, a diffuser in the bottom part, and an online spectrophotometer (257 nm). The reactor’s discharge was marked by the placement of a catalytic ozone destroyer tank. There is a prior paper that provides detailed specs for this reactor^29^.

### Data description

To construct a trustworthy ML model, precise and consistent data collection is crucial. Therefore, meticulous experimental records and conditions documented by K. S. Baig^29^ were utilized in this study. Although these data were obtained from laboratory-scale experiments conducted nearly a decade ago, they remain highly valuable for understanding the fundamental mechanisms of lignin removal through ozonation. The controlled experimental setup provides a reliable baseline for model development and validation. Moreover, the patterns, correlations, and predictive insights derived from this dataset offer guiding significance for scaling the ozonation process to industrial applications. Such ML-based modeling can help optimize process parameters, reduce experimental costs, and inform pilot-scale studies by identifying efficient operational ranges before full-scale implementation.

Time, flow rate ($\hbox {mg min}^{-1}$), percentage of lignin removed, O_3_ ($\hbox {mg min}^{-1}$) supplied, and L-unreacted ($\hbox {mg g}^{-1}$) were the crucial parameters in understanding lignin removal during ozonation. Time represents the experimental duration, influencing lignin degradation alongside flow rate, which affects ozone lignin interaction. The primary outcome, percentage of lignin removed, reflects treatment effectiveness. O_3_ supplied quantifies the ozone input, essential for evaluating its role in lignin breakdown. Conversely, L-unreacted ($\hbox {mg g}^{-1}$) indicates the remaining lignin, providing insights into treatment efficiency and optimization potential^30,31^.

### Data splitting into train/test

A common problem in modelling is that the models often perform well on data sets for training but poorly on fresh ones. To prevent this issue, known as overfitting, the dataset was divided into two parts: the training set and the testing set^32^. To create the model, one uses the training dataset, and to check its accuracy, one uses the test dataset. When that happens, the hyperparameters are changed and the model is retrained. We will keep doing this until our test datasets achieve high accuracy. The training set and test set are often 80:20 splits of the dataset^28^.

### Predictive models

A new technology that is emerging in this period is AI. In a nutshell, AI uses values from existing data, creates rules to apply to new, unknown data, and then generates predictions^33^. Using a combination of multiple regression approaches, this work engages ML accurately in a non-linear model^34^. The data used in this analysis come from experiments that utilized multiple variables as inputs. The data being analyzed was well-suited to a straightforward non-linear regression model technique. Rather than relying on basic regression, nonlinearity was addressed by employing a range of conventional ML regression approaches that consider numerous nonlinear inputs^35^.

ML models were being compared using Pycaret in Python programming. All the models that were examined showed a considerable improvement in accuracy when Pycaret was used. The variety of high-quality visualization capabilities provided by Pycaret made it easy to examine results and enhance models. One method for determining if attributes were linearly related is to utilize the pair plot^36,37^.

**Fig. 11:**
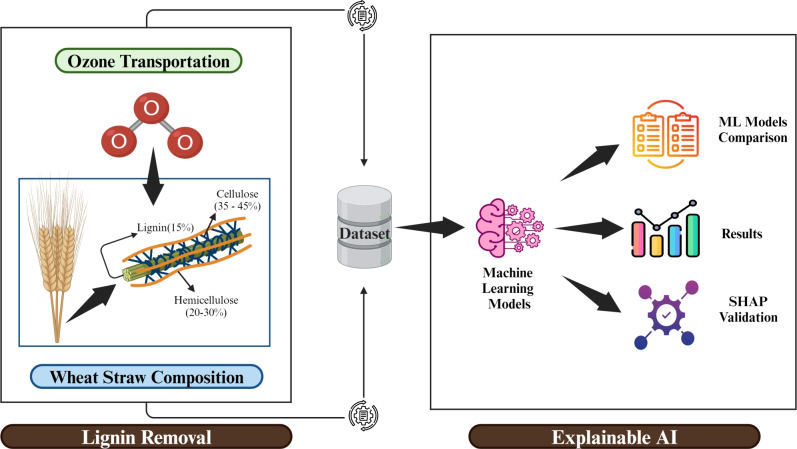
Proposed methodology.

ML methods aim to simplify experimental research by facilitating the understanding of variable correlations and the provision of highly accurate result predictions^38^. Experimental research based on trial and error was inefficient since it took a lot of time and cost a lot of money^39^. The time and money spent developing a set of experiments to conclude a research study was one of the major gaps in experimental research. That is why several methods have emerged, and why ML is quickly replacing human decision-makers in many contexts^40^. Data scientists have created a framework, ML, to efficiently mine, sort, and choose data, as well as to forecast outcomes and occurrences^41^. Because it is so versatile, ML has found a home in every scientific discipline. ML differs significantly from conventional computing techniques, which rely solely on pre-programmed instructions and do not incorporate any learning whatsoever. ML expedites and improves the accuracy of decision-making when used with a versatile learning platform^42,43^. Figure 11 shows the proposed methodology.

To ensure that each model fits its target, a 10-fold cross-validation procedure is employed. After that, we finalize and proceed with hyperparameter optimization for the top four models based on their weighted average accuracy metrics^44^.

### SHapley Additive exPlanations (SHAP)

By utilizing SHAP techniques, the model’s predictions were rendered more interpretable. Using the SHAP method, quantify the contribution of each feature to individual predictions was quantified, giving a complete understanding of how various factors impacted the model’s outputs^45^. Using concepts from game theory, the SHAP method shows how feature value contributes to increasing or decreasing goal value. By offering a more complex picture of the relationships recorded by the models, this interpretative tool helps to illuminate the prediction mechanisms and encourages transparency^46^.

In order to understand how characteristics, affect a given prediction, SHAP values represent the calculated average marginal impact for each feature over all possible combinations. The results of ML models may be better understood using this approach, which is grounded in cooperative game theory. Features are seen “players” in the framework, the reduction framework, which “contribute” to the final product. The final tally of all characteristics made up the model’s prediction results. As a function of the input vector *x*, the original predictive model’s output is provided by Eq.1:

1 $$\begin{aligned} f(x) = g(x') = \phi _0 + \sum _{i=1}^{M} \phi _i x_i \end{aligned}$$

where $f(\textit{x})$ is a high-dimensional feature vector and $g(\textit{x}')$ is a reduced or lower-dimensional version of $\textit{x}$ that approximates $f(\textit{x})$ and may be used as an interpretable surrogate model. The reason *g* is more standard than *f* is that it is frequently transformed into $\textit{x}'$ using feature selection or dimensionality reduction techniques. The parameter $\phi _0$ is used as a starting point for adding or subtracting the contributions of various features and $\phi _i$ is the SHAP value for the $i^{\text {th}}$ feature shown in Eq.2:

2 $$\begin{aligned} \phi _i = \sum _{B \subseteq N \setminus \{i\}} \frac{|B|! \, (|N| - |B| - 1)!}{|N|!} \left[ f(B \cup \{i\}) - f(B) \right] \end{aligned}$$

This is where $\phi _i$ stands for the Shapley value, which represents the feature *i*’s contribution to the prediction; *N* represents the whole set of features, including all items in the feature vector $\textit{x}$; and *B* is a subset of *N* that contains features chosen from the original feature vector $\textit{x}$. The parts of the characteristics represented by *i* are not in the subset *B*; the prediction function using the features in *B* is *f*(*B*). The term $\frac{|B|!(|N|-|B|-1)!}{|N|!}$ denotes the number of possible combinations that contain feature *i* and serves as the weighting factor.

A feature’s impact on a sample’s prediction outcome is greater for features with bigger absolute Shapley values. The significance and direction of each feature’s effect on a specific prediction may be understood by looking at the magnitude and sign of these Shapley values. In particular, if the Shapley value is positive (negative), it means that the related information helps the model anticipate a positive (negative) value.

By providing a unified picture across all samples, SHAP enhances the model’s global interpretability, allowing researchers to see the importance of each feature. Individuals can assess the feature’s relative relevance and its impact on model predictions by examining its SHAP value distribution. Features that consistently exhibit positive or negative absolute SHAP values show a systematic increase or decrease in the model’s prediction; accordingly, variables with larger absolute SHAP values are often more critical. Thus, the possible interaction between features and nonlinear dependencies may be better identified because of SHAP’s global interpretability.

### Evaluation metrics

To evaluate the efficacy of the input dataset for prediction, this study computed several widely used performance indicators^47^. These metrics were used to compare the predicted and actual values of the target variable (radon concentration in this case), allowing quantitative assessment of model accuracy and robustness. The performance was assessed using R-squared ($R^2$), Root Mean Square Error (RMSE), Root Mean Squared Log Error (RMSLE), Mean Squared Error (MSE), and Mean Absolute Percentage Error (MAPE)^48^. Each of these indicators provides different insights into the predictive capability and error characteristics of the model.

#### Root Mean Square Error (RMSE)

RMSE measures the average magnitude of prediction errors and indicates how concentrated the data is around the best-fit line. A smaller RMSE value suggests a better-fitting model. It is computed as shown in Eq. 3^49^:

3 $$\begin{aligned} \text {RMSE} = \sqrt{\frac{1}{V} \sum _{n=1}^{V} (X_n - Y_n)^2} \end{aligned}$$

where $V$ represents the total number of samples, $X_n$ denotes the actual observed value, and $Y_n$ is the predicted value.

#### Root Mean Squared Logarithmic Error (RMSLE)

RMSLE reduces the influence of large outliers and is useful when the target values vary across several orders of magnitude. It penalizes underestimations more than overestimations, making it suitable for skewed data distributions. The RMSLE is calculated as follows in Eq.‘4^50^:

4 $$\begin{aligned} \text {RMSLE} = \sqrt{\frac{1}{V} \sum _{n=1}^{V} \left( \log (X_n + 1) - \log (Y_n + 1) \right) ^2} \end{aligned}$$

#### Mean Absolute Error (MAE)

Mean Absolute Error (MAE) measures the average magnitude of the absolute differences between predicted and actual values, without considering their direction. It provides a straightforward measure of how close the predictions are to the actual observations. A smaller MAE value indicates higher model accuracy. It is calculated as shown in Eq. 5^51^:

5 $$\begin{aligned} \text {MAE} = \frac{1}{V} \sum _{n=1}^{V} |X_n - Y_n | \end{aligned}$$

#### Mean Absolute Percentage Error (MAPE)

MAPE expresses prediction accuracy as a percentage, making it scale-independent and easy to interpret. However, it can become unstable when actual values approach zero. The MAPE is given by Eq. 6^52^:

6 $$\begin{aligned} \text {MAPE} = \frac{1}{V} \sum _{n=1}^{V} \left| \frac{Y_n - X_n}{X_n} \right| \end{aligned}$$

#### Mean Squared Error (MSE)

MSE quantifies the average squared difference between actual and predicted values. It is particularly sensitive to large deviations, which makes it valuable for detecting models with high variance. The formula for MSE is presented in Eq. 7^53^:

7 $$\begin{aligned} \text {MSE} = \frac{1}{V} \sum _{n=1}^{V} (Y_n - X_n)^2 \end{aligned}$$

#### Coefficient of determination ($R^2$)

$R^2$ indicates how well the independent variables explain the variability of the dependent variable. A higher $R^2$ value (closer to 1) signifies that the model explains most of the variability in the data. The $R^2$ is computed using Eq. 8^54^:

8 $$\begin{aligned} R^2 = 1 - \frac{\frac{1}{n} \sum _{i=1}^{n} (y_i - \hat{y}_i)^2}{\frac{1}{n} \sum _{i=1}^{n} (y_i - \bar{y})^2} \end{aligned}$$

where $y_i$ represents the actual values, $\hat{y}_i$ are the predicted values, and $\bar{y}$ is the mean of the actual values.

These performance metrics collectively provide a comprehensive evaluation of the model’s predictive performance by measuring both absolute and relative errors, as well as the overall fit between actual and predicted values.

### Optimization workflow

To establish an optimized ozonation process for lignin removal, a systematic ML–based workflow was implemented. The PyCaret framework was used to train and compare 19 regression algorithms using 10-fold cross-validation. The best-performing model, Extra Trees Regressor, was further optimized through automated hyperparameter tuning (grid and Bayesian search). SHAP analysis quantified the contribution of input features, allowing identification of the most influential variables and their optimal operating ranges. The resulting workflow provides a data-driven optimization strategy for maximizing delignification efficiency with minimal experimental iterations.

## Conclusion

This study demonstrates the potential of ML in optimizing lignin extraction from lignocellulosic biomass using ozonation. By employing a comparative analysis of 19 classical ML models, it has been identified Extra Trees Regressor as the most accurate predictor of delignification. Extra trees regressor predicted accurately the delignification with the lowest deviation from the actual values such as (MAE = 0.7542, MSE = 0.9400, RMSE = 0.9070, $R^2$ = 0.9874, RMSLE = 0.0117, MAPE = 0.0100). The application of SHAP enabled a deeper understanding of feature importance, providing insights into the extraction process. Our findings lay the groundwork for the development of efficient and selective lignin removal processes, which could significantly impact the production of biofuels, chemicals, and materials. This approach can be modified for use in future studies to address more delignification problems in chemical engineering. The accuracy of predictions might also be enhanced by investigating more complex neural networks. Improving the practical application of ML in chemical engineering might be made easier with tailor-built interpretability frameworks. This would allow the creation of AI applications for complex engineering systems that are more visible, responsible, and verifiable.

## Data Availability

Data and code available on https://doi.org/10.6084/m9.figshare.29289128.v1

## References

[1] Allahyarzadeh-Bidgoli, A., Dezan, D. J., Salviano, L. O., de Oliveira Junior, S. & Yanagihara, J. I. Fpso fuel consumption and hydrocarbon liquids recovery optimization over the lifetime of a deep-water oil field. *Energy* **181**, 927–942. 10.1016/j.energy.2019.05.146 (2019).

[2] Oumer, A. N., Hasan, M. M., Baheta, A. T., Mamat, R. & Abdullah, A. A. Bio-based liquid fuels as a source of renewable energy: A review. *Renew. Sustain. Energy Rev.* **88**, 82–98. 10.1016/j.rser.2018.02.022 (2018).

[3] Babu, S. et al. Exploring agricultural waste biomass for energy, food and feed production and pollution mitigation: A review. *Bioresour. Technol.* **360**, 127566. 10.1016/j.biortech.2022.127566 (2022). 35788385 10.1016/j.biortech.2022.127566

[4] Hatakeyama, H. & Hatakeyama, T. *Lignin Structure, Properties, and Applications, 1–63* (Springer, 2010).

[5] Mendu, V. et al. Global bioenergy potential from high-lignin agricultural residue. *Proc. Natl. Acad. Sci. United States Am.* **109**, 4014–4019. 10.1073/pnas.1112757109 (2012). 10.1073/pnas.1112757109 PMC3309743 22355123

[6] Beig, B. et al. Current challenges and innovative developments in pretreatment of lignocellulosic residues for biofuel production: A review. *Fuel* **287**, 119670. 10.1016/j.fuel.2020.119670 (2021).

[7] Taherzadeh, M. J. & Karimi, K. Pretreatment of lignocellulosic wastes to improve ethanol and biogas production: A review. *Int. J. Mol. Sci.* **9**, 1621–1651. 10.3390/ijms9091621 (2008). 19325822 10.3390/ijms9091621 PMC2635757

[8] Khullar, E., Dien, B. S., Rausch, K. D., Tumbleson, M. E. & Singh, V. Effect of particle size on enzymatic hydrolysis of pretreated miscanthus. *Ind. Crop. Prod.* **44**, 11–17. 10.1016/j.indcrop.2012.10.015 (2013).

[9] Baksi, S. et al. Pre-treatment of lignocellulosic biomass: review of various physico-chemical and biological methods influencing the extent of biomass depolymerization. *Int. J. Environ. Sci. Technol.* **20**, 13895–13922. 10.1007/s13762-023-04838-4 (2023).

[10] Dussán, K. J., Silva, D. D. V., Costa, A. F. M., Grangeiro, L. C. & Giese, E. C. *Downstream Processing in Lignocellulose Conversion*, 261–288 (Wiley Online Library, 2022). Wiley Online Books.

[11] Badiei, M., Asim, N., Jahim, J. M. & Sopian, K. Comparison of chemical pretreatment methods for cellulosic biomass. *APCBEE Procedia* **9**, 170–174. 10.1016/j.apcbee.2014.01.030 (2014).

[12] Akpan, E. I., Wetzel, B. & Friedrich, K. Eco-friendly and sustainable processing of wood-based materials. *Green Chem.* **23**, 2198–2232. 10.1039/D0GC04430J (2021).

[13] Wu, Z. et al. Lignocellulose dissociation with biological pretreatment towards the biochemical platform: A review. *Mater. Today Bio* **16**, 100445. 10.1016/j.mtbio.2022.100445 (2022). 36212906 10.1016/j.mtbio.2022.100445 PMC9535326

[14] Wei, C., Zhang, F., Hu, Y., Feng, C. & Wu, H. Ozonation in water treatment: the generation, basic properties of ozone and its practical application. *Rev. Chem. Eng.* **33**, 49–89. 10.1515/revce-2016-0008 (2017).

[15] Nges, I. A. et al. Physio-chemical pretreatments for improved methane potential of miscanthus lutarioriparius. *Fuel* **166**, 29–35. 10.1016/j.fuel.2015.10.108 (2016).

[16] Strielkowski, W., Vlasov, A., Selivanov, K., Muraviev, K. & Shakhnov, V. Prospects and challenges of the machine learning and data-driven methods for the predictive analysis of power systems: A review. *Energies*10.3390/en16104025 (2023).

[17] Yan, Y., Borhani, T. N. & Clough, P. T. *Machine Learning Applications in Chemical Engineering*, 0 (The Royal Society of Chemistry, 2020).

[18] Muqeet, M. et al. Enhanced cellulose nanofiber mechanical stability through ionic crosslinking and interpretation of adsorption data using machine learning. *Int. J. Biol. Macromol.* **237**, 124180. 10.1016/j.ijbiomac.2023.124180 (2023). 36990398 10.1016/j.ijbiomac.2023.124180

[19] Guo, Y., Yang, M., Huang, G. & Zheng, Y. Machine-learning-enabled exploitation of gas-sensing descriptors: A case study of five pristine metal oxides. *Chem. Eng. J.* **492**, 152280 (2024).

[20] Rohani, V. A., Peerally, J. A., Moghavvemi, S., Guerreiro, F. & Pinho, T. Illustrating scholar–practitioner collaboration for data-driven decision-making in the optimization of logistics facility location and implications for increasing the adoption of ar and vr practices. *The TQM J.* **34**, 280–302. 10.1108/TQM-06-2021-0194 (2022).

[21] Keith, J. A. et al. Combining machine learning and computational chemistry for predictive insights into chemical systems. *Chem. Rev.* **121**, 9816–9872. 10.1021/acs.chemrev.1c00107 (2021). 34232033 10.1021/acs.chemrev.1c00107 PMC8391798

[22] Zhang, T. et al. Machine learning prediction of photocatalytic lignin cleavage of c–c bonds based on density functional theory. *Mater. Today Sustain.* **20**, 100256. 10.1016/j.mtsust.2022.100256 (2022).

[23] Baran, K., Barczak, B. & Kloskowski, A. Modeling lignin extraction with ionic liquids using machine learning approach. *Sci. The Total. Environ.* **935**, 173234. 10.1016/j.scitotenv.2024.173234 (2024). 10.1016/j.scitotenv.2024.173234 38768717

[24] Hu, J. et al. Explainable ai models for predicting drop coalescence in microfluidics device. *Chem. Eng. J.* **481**, 148465. 10.1016/j.cej.2023.148465 (2024).

[25] König-Mattern, L. et al. Machine learning-supported solvent design for lignin-first biorefineries and lignin upgrading. *Chem. Eng. J.* **495**, 153524. 10.1016/j.cej.2024.153524 (2024).

[26] Stančin, I. & Jović, A. An overview and comparison of free python libraries for data mining and big data analysis. In *2019 42nd International Convention on Information and Communication Technology, Electronics and Microelectronics (MIPRO)*, 977–982, 10.23919/MIPRO.2019.8757088 (2019).

[27] Kartal, F. & Özveren, U. An improved machine learning approach to estimate hemicellulose, cellulose, and lignin in biomass. *Carbohydr. Polym. Technol. Appl.* **2**, 100148. 10.1016/j.carpta.2021.100148 (2021).

[28] Ahmad, M. B., Soomro, U., Muqeet, M. & Ahmed, Z. Adsorption of indigo carmine dye onto the surface-modified adsorbent prepared from municipal waste and simulation using deep neural network. *J. Hazard. Mater.* **408**, 124433. 10.1016/j.jhazmat.2020.124433 (2021). 33257121 10.1016/j.jhazmat.2020.124433

[29] Baig, K. S., Wu, J., Turcotte, G. & Doan, H. D. Novel ozonation technique to delignify wheat straw for biofuel production. *Energy & Environ.* **26**, 303–318. 10.1260/0958-305X.26.3.303 (2015).

[30] Baig, K. S. Kinetics of lignin removal from the lignocellulosic matrix after ozone transportation. *Methane* **1**, 177–188. 10.3390/methane1030014 (2022).

[31] Atiwesh, G., Parrish, C. C., Banoub, J. & Le, T.-A.T. Lignin degradation by microorganisms: A review. *Biotechnol. Prog.* **38**, e3226. 10.1002/btpr.3226 (2022). 34854261 10.1002/btpr.3226

[32] Salazar, J. J., Garland, L., Ochoa, J. & Pyrcz, M. J. Fair train-test split in machine learning: Mitigating spatial autocorrelation for improved prediction accuracy. *J. Petroleum Sci. Eng.* **209**, 109885. 10.1016/j.petrol.2021.109885 (2022).

[33] Borges, A. F. S., Laurindo, F. J. B., Spínola, M. M., Gonçalves, R. F. & Mattos, C. A. The strategic use of artificial intelligence in the digital era: Systematic literature review and future research directions. *Int. J. Inf. Manag.* **57**, 102225. 10.1016/j.ijinfomgt.2020.102225 (2021).

[34] Krmar, J. et al. Performance comparison of nonlinear and linear regression algorithms coupled with different attribute selection methods for quantitative structure - retention relationships modelling in micellar liquid chromatography. *J. Chromatogr. A* **1623**, 461146. 10.1016/j.chroma.2020.461146 (2020). 32505269 10.1016/j.chroma.2020.461146

[35] Mizumoto, A. Calculating the relative importance of multiple regression predictor variables using dominance analysis and random forests. *Lang. Learn.* **73**, 161–196. 10.1111/lang.12518 (2023).

[36] Whig, P. et al. A novel method for diabetes classification and prediction with pycaret. *Microsyst. Technol.* **29**, 1479–1487. 10.1007/s00542-023-05473-2 (2023).

[37] James, G., Witten, D., Hastie, T., Tibshirani, R. & Taylor, J. *Linear Regression*, 69–134 (Springer International Publishing, 2023).

[38] Zhu, J.-J., Yang, M. & Ren, Z. J. Machine learning in environmental research: Common pitfalls and best practices. *Environ. Sci. & Technol.* **57**, 17671–17689. 10.1021/acs.est.3c00026 (2023). 37384597 10.1021/acs.est.3c00026

[39] Schmidt, M. et al. Real-time, wide-field, and quantitative oxygenation imaging using spatiotemporal modulation of light. *J. Biomed. Opt.* **24**, 071610. 10.1117/1.JBO.24.7.071610 (2019). 30868804 10.1117/1.JBO.24.7.071610 PMC6995963

[40] Duan, Y., Edwards, J. S. & Dwivedi, Y. K. Artificial intelligence for decision making in the era of big data – evolution, challenges and research agenda. *Int. J. Inf. Manag.* **48**, 63–71. 10.1016/j.ijinfomgt.2019.01.021 (2019).

[41] Sarker, I. H. Machine learning: Algorithms, real-world applications and research directions. *SN Comput. Sci.* **2**, 160. 10.1007/s42979-021-00592-x (2021). 33778771 10.1007/s42979-021-00592-x PMC7983091

[42] Tien, J. M. Internet of things, real-time decision making, and artificial intelligence. *Annals Data Sci.* **4**, 149–178. 10.1007/s40745-017-0112-5 (2017).

[43] Ezugwu, A. E. et al. A comprehensive survey of clustering algorithms: State-of-the-art machine learning applications, taxonomy, challenges, and future research prospects. *Eng. Appl. Artif. Intell.* **110**, 104743. 10.1016/j.engappai.2022.104743 (2022).

[44] Nti, I. K., Nyarko-Boateng, O. & Aning, J. Performance of machine learning algorithms with different k values in k-fold crossvalidation. *Int. J. Inf. Technol. Comput. Sci.* **13**, 61–71. 10.5815/ijitcs.2021.06.05 (2021).

[45] Khan, M. A., Khan, M., Dawood, H., Dawood, H. & Daud, A. Secure explainable-ai approach for brake faults prediction in heavy transport. *IEEE Access* **12**, 114940–114950. 10.1109/ACCESS.2024.3444907 (2024).

[46] Clark, M. P. et al. A unified approach for process-based hydrologic modeling: 1. modeling concept. *Water Resour. Res.* **51**, 2498–2514. 10.1002/2015WR017198 (2015).

[47] Myrtveit, I., Stensrud, E. & Olsson, U. H. Analyzing data sets with missing data: an empirical evaluation of imputation methods and likelihood-based methods. *IEEE Transactions on Softw. Eng.* **27**, 999–1013. 10.1109/32.965340 (2001).

[48] Papenmeier, A., Kern, D., Englebienne, G. & Seifert, C. It’s complicated: The relationship between user trust, model accuracy and explanations in ai. *ACM Transactions on Comput. Interact.*10.1145/3495013 (2022).

[49] Hodson, T. O. Root-mean-square error (rmse) or mean absolute error (mae): when to use them or not. *Geosci. Model. Dev.* **15**, 5481–5487. 10.5194/gmd-15-5481-2022 (2022).

[50] Efron, B. Estimation and accuracy after model selection. *J. Am. Stat. Assoc.* **109**, 991–1007. 10.1080/01621459.2013.823775 (2014). 25346558 10.1080/01621459.2013.823775 PMC4207812

[51] Hodson, T. O. Root mean square error (rmse) or mean absolute error (mae): When to use them or not. *Geosci. Model. Dev. Discuss.* **2022**, 1–10 (2022).

[52] Chicco, D., Warrens, M. J. & Jurman, G. The coefficient of determination r-squared is more informative than smape, mae, mape, mse and rmse in regression analysis evaluation. *PeerJ Comput. Sci.* **7**, e623. 10.7717/peerj-cs.623 (2021). 34307865 10.7717/peerj-cs.623 PMC8279135

[53] Marmolin, H. Subjective mse measures. *IEEE Transactions on Syst. Man, Cybern.* **16**, 486–489. 10.1109/TSMC.1986.4308985 (1986).

[54] Miles, J. *R-Squared, Adjusted R-Squared*, 1234–8 (Wiley Online Library, 2005). Major Reference Works.

